# The first case report of thorn-induced *Alternaria alternata* infection of the hand in an immunocompetent host

**DOI:** 10.1186/s12879-022-07280-y

**Published:** 2022-03-29

**Authors:** Gyeongmin Kim, Seung Jin Yoo, Jeong Rae Yoo, Kyu Bum Seo

**Affiliations:** 1 Division of Hand Surgery, Department of Orthopedic Surgery, Jeju National University Hospital, Jeju, South Korea; 2 Division of Infectious Disease, Department of Internal Medicine, Jeju National University Hospital, Jeju, South Korea

**Keywords:** *Alternaria alternata*, Fungal infection, *Thorn injury*, Extensor tenosynovitis, Septic arthritis

## Abstract

**Background:**

Fungal infection secondary to a penetrating plant thorn injury in upper extremities is infrequently reported especially in immunocompetent hosts. *Alternaria* is a dematicaceous hyphomycete, commonly found in decay and plant pathogens, and *Alternaria alternata* has been regarded as the most frequent species among more than 400 s of its species. This case is the first report of thorn-induced *Alternaria alternata* infection of the hand in an immunocompetent host.

**Case presentation:**

A 47-year-old male patient was admitted to our institution with persistent pain and swelling of the right hand even after a prior surgical removal of a previous thorn injury. Upon impression of abscess, chronic extensor tenosynovitis, and septic arthritis at the 3rd metacarpophalangeal joint based on advanced imaging, the patient underwent surgical incision and drainage. Intraoperative culture, biopsy, and gene molecular sequencing results revealed fungal infection with *Alternaria alternata.* Postoperatively, the patient was treated with oral itraconazole (200 mg q 12 h) for nine consecutive months.

**Conclusions:**

We report the first case of chronic extensor tenosynovitis and septic arthritis of the hand with *Alternaria alternata* after a thorn injury in an immunocompetent host. Despite rare incidences of fungal extensor tenosynovitis and septic arthritis, the current case strongly suggests a careful examination of social history and surgical debridement along with a prolonged use of appropriate anti-fungal agents in thorn injuries.

## Background

Fungal infection, involving tenosynovium and joints, secondary to penetrating plant thorn injury is infrequently reported especially in immunocompetent hosts. [1] Opportunistic cutaneous and subcutaneous fungal infection by *Alternaria species* has been reported in the previous literature but mostly among the immunocompromised hosts, whose risk factors include hematologic malignancies, diabetes, autoimmune diseases, acquired immunodeficiency or a prolonged use of corticosteroids. [2–5] *Alternaria* is a dematiaceous hyphomycete, commonly found in decay and plant pathogens and frequently associated with hypersensitivity pneumonitis, asthma, allergic sinusitis and rhinitis, and *Alternaria alternata* has been regarded as the most frequent species among more than 400 s of its species. [6].

To our knowledge, we report the first case of thorn-induced *Alternaria alternata* infection of the hand causing chronic extensor tenosynovitis, abscess, and septic arthritis in a healthy, immunocompetent, and young male patient, who was successfully treated with surgical debridement and a prolonged use of oral anti-fungal agent.

## Case presentation

**Fig. 1:**
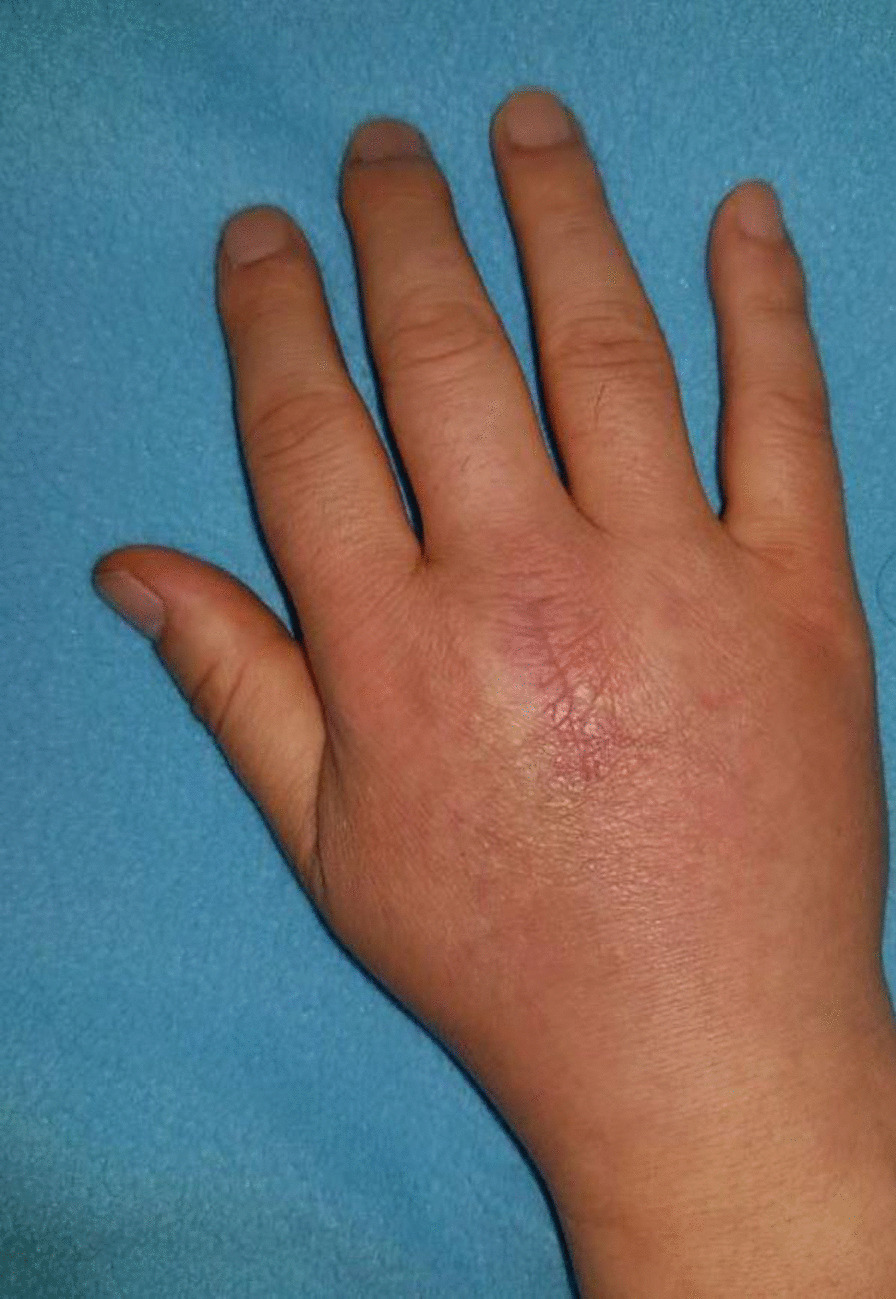
Preoperative assessment of the affected hand. The right hand indicates previous operative scar at the 3rd metacarpophalangeal (MCP) joint with mild erythema and swelling on dorsal aspect of the hand

**Fig. 2:**
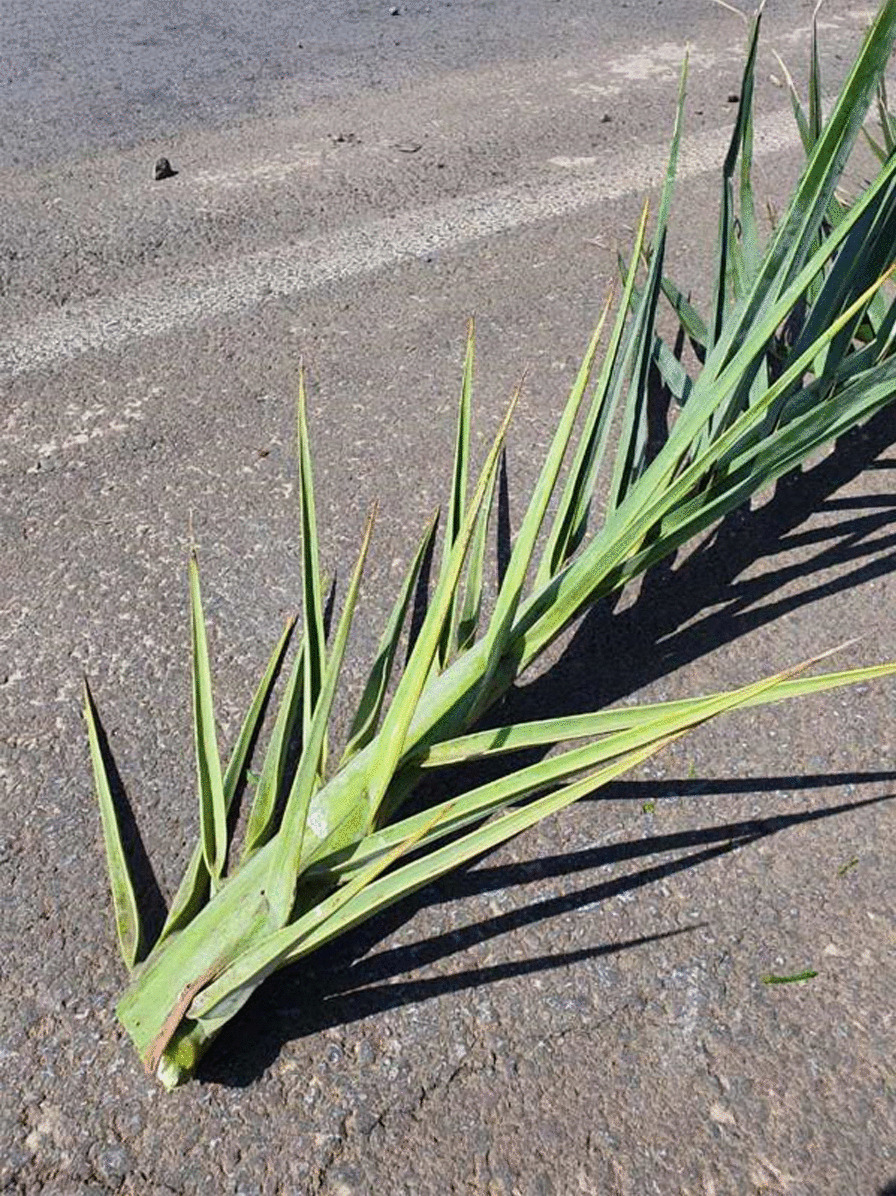
Thorn injury by a palm tree. The patient had experienced the thorn injury while pruning a palm tree 12 months prior to the admission

A 47-year-old male patient was admitted to the department of orthopedic surgery with a chief complaint of persistent pain and swelling on the right hand for the past two months. (Fig. 1) The patient had experienced a thorn injury at a dorsal aspect of the hand while pruning a palm tree 12 months ago, and the thorn was surgically removed at another institution 5 months prior to the visit to our institution. (Fig. 2) Postoperatively, the patient had routine oral antibiotics (cephalexin 500 mg q 8 h) treatments for 7 days. The patient reported that he had not had any clinical symptoms of pain, swelling, and tenderness until the one month before the first surgery.

The patient, whose occupation was a solider of Republic of Korea marine Corps, did not have previous underlying diseases or a history of prior fungal infection. Physical examination indicated stable vital sign with focal tenderness, swelling, and mild heating sense over the dorsal aspect of 3rd metacarpophalangeal (MCP) joint area without cutaneous lesions. The patient did not show any evidence of initiating pain during flexion and extension of the 3rd finger. Initial laboratory findings, including white blood cell counts, eosinophil counts, C-reactive protein, and erythrocyte sedimentation rate, were all in the normal range. Human immunodeficiency virus was negative.

**Fig. 3:**
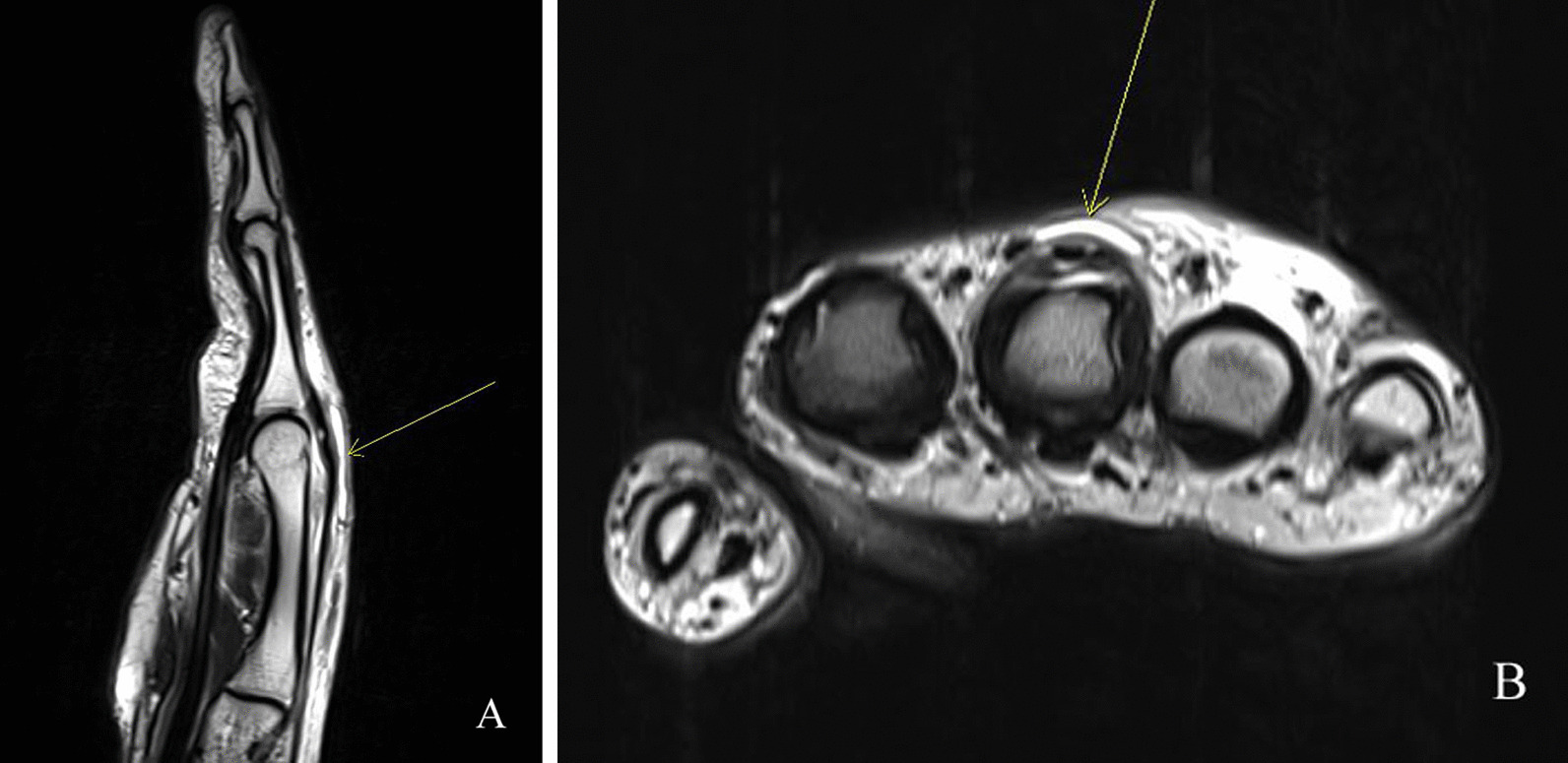
Preoperative enhanced magnetic resonance imaging of the hand. A thin elongated fluid collection at the dorsal aspect of 3rd finger MCP joint level, enhancement along the extensor tendon of 3rd finger, joint effusion with thick enhancing synovium at 3rd MCP joint suggest abscess, chronic extensor tenosynovitis, and septic arthritis, respectively, on gadolinium-enhanced T2-weighted sagittal (**A**) and axial (**B**) images. Yellow arrow in both (**A**) and (**B**) indicates 3rd MCP joint

**Fig. 4:**
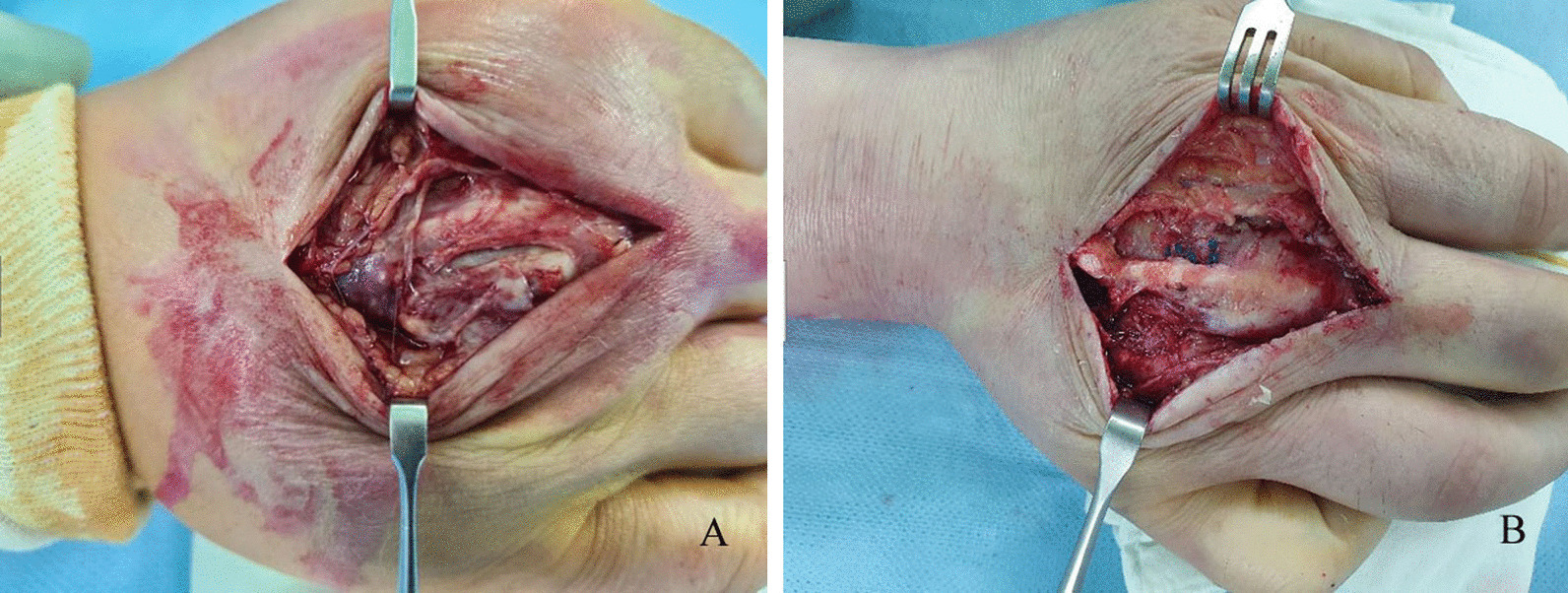
Intraoperative images after tenosynovectomy and debridement.** (A)** severe adhesion of soft tissue with granulation and infective tissues and partial rupture at 3rd MCP joint capsule (**B)** post-tenosynovectomy and debridement with primary repair of ruptured 3rd MCP joint capsule

**Fig. 5:**
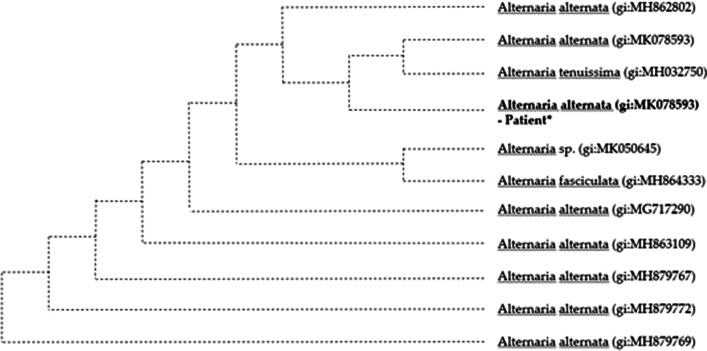
Phylogenetic tree constructed based on internal transcribed spacer (ITS) gene sequence of *Alternaria alternata*. Gene sequencing analysis of ITS gene, amplified with the primer pair ITS1 (5′-TCCGTAGGTGAACCTGCGG-3′) and ITS4 (5′TCCTCCGCTTATTGATATGC)-3′, indicated *Alternaria alternata*. *Alternaria alternata* was confirmed with an 100% accuracy using the basic local alignment search tool algorithm. (*, patient). Scale bar indicated nucleotide substitutions per site

Enhanced magnetic resonance imaging (MRI) of the right hand indicated a thin elongated fluid collection at the dorsal aspect of 3rd finger MCP joint level, enhancement along the extensor tendon of 3rd finger, joint effusion with thick enhancing synovium at 3rd MCP joint, suggesting abscess, chronic extensor tenosynovitis, and septic arthritis, respectively (Fig. 3A and B). Consequently, emergent surgical incision and drainage was planned upon impression of chronic extensor tenosynovitis, abscess, and septic arthritis at the 3rd MCP joint. Intraoperative findings revealed severe adhesion of soft tissue with granulation and infective tissues and partial rupture at 3rd MCP joint capsule (Fig. 4A and B). Meticulous debridement and massive irrigation were performed along with intraoperative tissue culture and biopsy. In addition, we performed fungus culture from tissue specimens on Sabouraud’s dextrose agar media plate (Asan Pharm. Co., LTD, Seoul, Korea), incubated at 30 °C. Bacterial culture, acid-fast bacilli stain, and tuberculosis-polymerase chain reaction were negative from the tissue specimens. On the eighth cultivating day, grayish soft fungal organisms were evident on the media. Microscopic examination of the cultured isolate with lactophenol cotton blue revealed an *Alternaria* species. Genomic DNA was extracted from fungal cells of the *Alternaria* species colonies. The primers used to amplify and sequence the nuclear ribosomal internal transcribed spacer (ITS) gene for fungus were ITS1, 5′-TCCGTAGGTGAACCTGCGG-3′ and ITS4, 5′TCCTCCGCTTATTGATATGC-3′. The fungus was identified as *Alternaria alternata* with 100% homology according to the BLAST program (www.ncbi.nlm.nih.gov/BLAST) and compared with the similar sequences in the Gene Bank [7]. A phylogenetic tree was constructed based on ITS gene sequences, using the maximum likelihood method implemented in MEGA 6 [8] (Fig. 5).

**Fig. 6:**
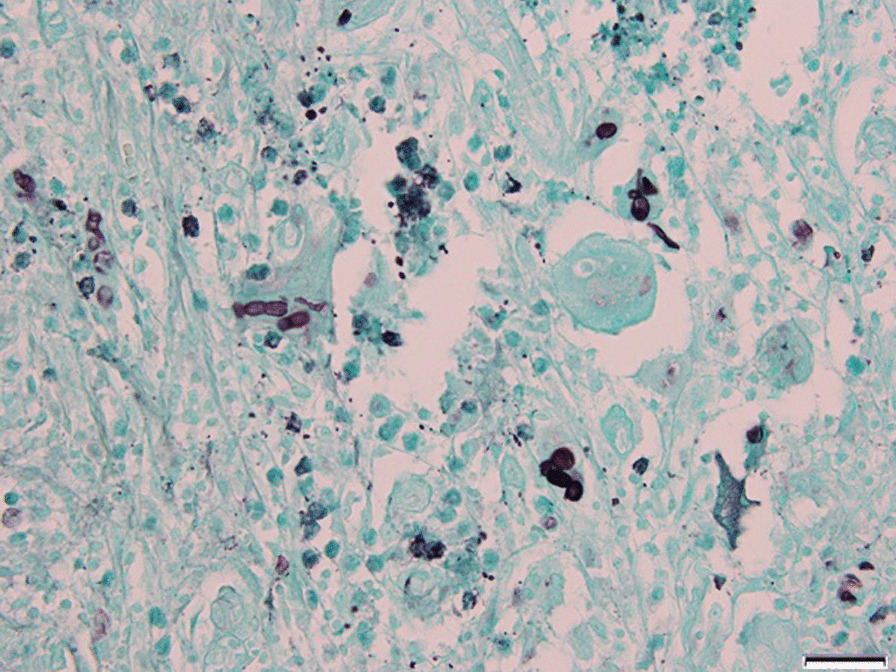
Histopathologic assessment. Chronic active inflammation with round-shaped fungal elements and multinucleated giant cells with positive Grocott Methenamine Silver stains (Magnification = 400X, Scale bar = 50 μm). Microscopic image was captured using Olympus BX53 light microscope, Olympus DP21 camera, and Olympus cellSens imaging software

Furthermore, histopathology indicated chronic active inflammation with round-shaped fungal elements and multinucleated giant cells with positive periodic acid Schiff and Grocott Methenamine Silver stains. (Fig. 6)

Postoperatively, the patient was treated with oral itraconazole (200 mg q 12 h) for 9 consecutive months. Itraconazole was well tolerated without any side-effects. At the end of the treatment with the anti-fungal agent, the patient was symptom-free with laboratory findings in normal ranges, indicating total resolution with no clinical relapse.

## Discussion and conclusion

*Alternaria alternata* is a dematiaceous fungus, primarily a plant pathogen and a saprophyte found in soil, air, and a variety of other habitats, including normal human skin and conjunctiva. [6, 9, 10] Fungal infection with *Alternaria alternata* is frequently associated with ocular infection, rhinosinusitis, onychomycosis, and cutaneous and subcutaneous infections. In the cutaneous and subcutaneous infection with *Alternaria alternata*, predisposing factors are associated with immunocompromised status, such as commonly organ transplantation, Cushing’s syndrome, and prolonged uses of immunosuppressive agents. Cutaneous and subcutaneous alternariosis shows a wide range of clinical manifestations, such as erythema, skin desquamation, papules, plaques, erosion, and ulceration. [6] *Alternaria *spp. infection associated with flexor tenosynovitis has been reported only once in a immunocompetent 6-year-old boy after a thorn injury in the previous literature. [11] To our knowledge, the current case is the first report of *Alternaria alternata* infection, involving extensor tenosynovium and a MCP joint, without cutaneous manifestation in an immunocompetent host.

Differential diagnosis of atypical tenosynovitis includes fungal infection, tuberculous and non-tuberculous mycobacterium infection, brucellosis, and rheumatoid arthritis. [12, 13] In fungal tenosynovitis, the median time from the development of symptoms to diagnosis was reported average 6 months, characterized by subacute worsening of symptoms, and advanced imaging modalities such as MRI or ultrasonography were essential preoperative diagnostic evaluation. Commonly identified fungal pathogens in hand infection include *Histoplasma, Coccidiodies*, and *Cryptococcus*. [13] Intraoperative bacterial, fungal, mycobacterial and histopathologic investigations are required to establish a diagnosis of specific pathogen and are critical to guiding treatment. Definitive diagnosis is made by positive fungal culture, but fungal cultures are often difficult to grow and may even take several weeks to show positive results; therefore, it is recommended to obtain multiple tissue samples for fungal culture intraoperatively. Infection caused by *Alternaria alternata* is uncommon, but its incidence is not well estimated due to the need for gene sequencing to identify the specific species of *Alternaria spp.* The current case report describes an immunocompetent and healthy patient with infection of subcutaneous, tenosynovial, and articular involvement, and fungal species was successfully cultured via intraoperative tissue culture and gene molecular sequencing not to delay subsequent oral anti-fungal therapy postoperatively.

There are no consensus or treatment guidelines for the treatment of fungal tenosynovitis. It generally requires extensive tenosynovectomy and debridement of all affected synovial tissues as well as a prolonged duration of anti-fungal therapy, and multiple consecutive debridement may be necessary depending on the severity of infection and degree of soft tissue integrity. Even though there is no formal recommendation for the anti-fungal regimen, the duration is typically from 3 to 12 months, considering the burden of the disease. In cases of osteoarticular infection, relapses, disseminated infection, or osteomyelitis, it may require a longer duration of anti-fungal treatment as long as 1 to 2 years. [13, 14] Itraconazole has been widely used and recommended in the treatment of cutaneous alternariosis, and amphotericin B is also accepted for *Alternaria* infection with a disadvantage of intravenous administration. [5, 15] In addition, voriconazole has also been reported in successful medical management of cutaneous alternariosis in immunocompromised hosts. [16].

In conclusion, to the best of our knowledge, fungal infection with *Alternaria alternata* involving chronic tenosynovitis and septic arthritis of hand has never been reported. *Alternaria alternata* should be considered in the differentials of atypical chronic tenosynovitis. Careful evaluation of patient’s history, including medical and occupational histories, is essential in diagnostic process of chronic tenosynovitis, and a history of even minor traumatic injuries including a thorn injury can be a critical diagnostic clue.

## Data Availability

The internal transcribed space of rRNA sequences of *Alternaria alternata* was available in the GenBank database (Accession Number: MK078593.1).

## Abbreviations

MCP: Metacarpophalangeal
MRI: Magnetic Resonance Imaging
ITS: Internal Transcribed Spacer
